# Oxidative degradation of alpha-tocopherol by reactive oxygen species, identifying products, and product anticancer activity

**DOI:** 10.1186/s13065-025-01665-1

**Published:** 2025-11-15

**Authors:** Hussein M. Ali, Mohamed H. Attia, Wael Mamdouh, Eman N. Rashed, Isra H. Ali

**Affiliations:** 1 Agricultural Biochemistry Department, Faculty of Agriculture, Ain-Shams University, Hadayek Shoubra, P.O. Box 68, Cairo, 11241 Egypt; 2 Department of Chemistry, School of Sciences and Engineering, The American University in Cairo (AUC), AUC Avenue, P.O. Box 74, New Cairo, 11835 Egypt; 3 Department of Pharmaceutics, Faculty of Pharmacy, University of Sadat City, Sadat City, 32897 Egypt; 4 Present Address: Department of Cardiovascular Medicine, Mayo Clinic, Rochester, MN USA

**Keywords:** Α-Tocopheryl quinone, Hydrogen peroxide, Hypochlorous acid, Oxidation products, Breast cancer, Prostate cancer

## Abstract

α-Tocopherol (α-TQ) is a potent antioxidant with diverse applications in the food and pharmaceutical industries. It is susceptible to oxidation by various reactive oxygen species (ROS). This study first identifies the oxidation product of α-tocopherol produced by either H_2_O_2_ or HOCl, which could be formed in foods and biological systems. Second, the kinetic and mechanistic aspects of these oxidation reactions are characterized. Finally, the anticancer activity of α-TQ and its oxidation products was revealed. The direct oxidation product is α-tocopheryl quinone (α-TQQ), which dimerizes through an ether linkage and undergoes addition reactions. LC-MS/MS identified new products, primarily including positional and diastereoisomers of α-TQQ dimers resulting from H_2_O_2_ and HOCl addition. A kinetic study demonstrated that the oxidation reaction is first-order for both α-TQ and H_2_O_2_ or HOCl. The mechanism is proposed based on the identified products and kinetic behavior. The postulated mechanism also aligns with the reaction’s UV-Vis spectra, including the formation of the hemiketal (242 nm) and α-TQQ (261 nm) intermediates, as well as the addition products of the α-TQQ dimer (265 nm). α-TQQ dimer products of H_2_O_2_ oxidation reaction exhibited 1.7-fold (IC50 264.72 µM) and 2.0-fold (IC50 253.72 µM) higher cytotoxicity than that of α-TQ (IC50 448.45 and 496.53 µM) against breast (MCF-7) and prostate (PC-3) cancer cells, respectively. These results indicate that natural α-TQ and its oxidation products, when administered at a suitable dose, can express protection against various types of cancer.

**Graphical abstract:**

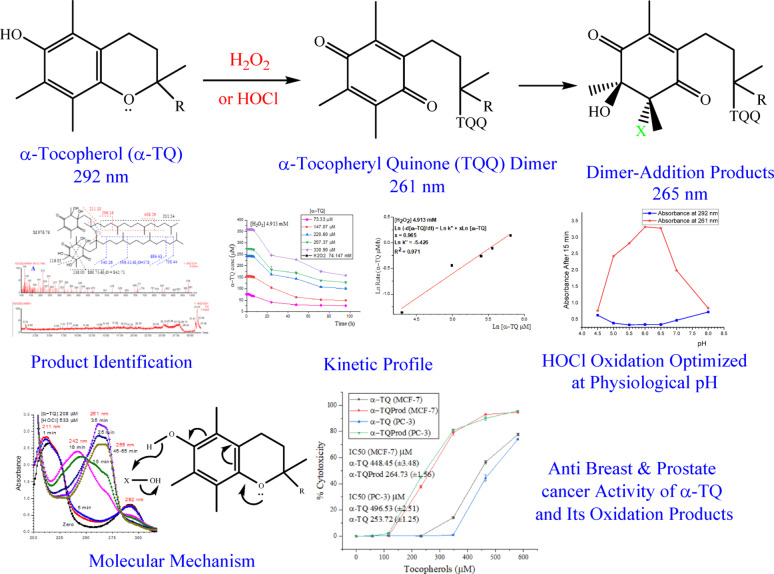

**Supplementary Information:**

The online version contains supplementary material available at 10.1186/s13065-025-01665-1.

## Highlights

- Oxidation of α-tocopherol (α-TQ) by H_2_O_2_ or HOCl gives α-tocopheryl quinone (α-TQQ).
- LC-MS/MS revealed that α-TQQ undergoes fast dimerization and H_2_O_2_ or HOCl addition.
- The oxidation is 1st order in each reactant. pH affects rate of α-TQ-HOCl reaction.
- α-TQ showed toxicity (IC50) for breast (448.45 µM) & prostate (496.53 µM) cancers.
- Dimer products gave 1.7 & 2.0-fold higher cytotoxicity (for both cancers) than α-TQ.

**Supplementary Information:**

The online version contains supplementary material available at 10.1186/s13065-025-01665-1.

## Introduction

Tocopherols (vitamin E) are the most common natural fat-soluble antioxidants mainly found in vegetable oils [1, 2]. Among the eight known forms of vitamin E (α, β, γ, and δ tocopherols and tocotrienols) α-tocopherol (α-TQ) is the most abundant and biologically active form existing in plasma membrane and tissues since it is also the most form found in human diets or provided as a supplement [3, 4]. The primary functions of α-tocopherol are its antioxidant activity and protection against lipid peroxidation induced by reactive oxygen species (ROS) [5, 6]. The potent antioxidant activity of α-tocopherol is attributed to its phenolic structure and the full electron-donating alkyl and alkoxy substitutions of the aromatic ring [7]. α-Tocopherol has diverse and significant health impacts, including cardiovascular [1], Alzheimer’s, Parkinson’s, and UV-induced skin diseases [3], as well as improving depression and anxiety in patients [8]. In addition, lipid peroxidation commonly takes place in foods during processing and storage, and thus, vitamin E is frequently used as a supplement to stabilize foods and extend their shelf life [3, 5, 9, 10]. However, the role of α-tocopherol is more complicated and not fully revealed; for example, although α-TQ was ineffective as an anticancer agent, other forms of vitamin E, tocotrienols [11], and the oxidation product of tocopherols, tocopheryl quinone (TQQ) [12], exhibited good activity. The activity of TQQ could be due to the activity of the conjugated double bonds [13, 14]. In addition, α-TQ acts as a pro-oxidant at high concentrations and under certain conditions; besides, it can alter the oil oxidation products [5, 15].

Reactive oxygen species (ROS) are active substances, either neutral or ionic, and radical or non-radical, produced naturally in biological systems from oxygen through the electron transfer process. They are responsible for many chronic diseases and food deterioration in addition to having considerable effects on environmental elements, e.g., soil, atmosphere, and water [16, 17]. Therefore, shedding light on their complex reactions in biological systems, identifying the oxidation products, and protecting them against oxidation are significant for healthcare and food production. Hydrogen peroxide (H_2_O_2_) is one of the primary formed ROS generated during some physiological processes, e.g., the action of some oxidases, such as xanthine, amino acid, and glucose oxidases [18, 19], the dismutation of superoxide anion by superoxide dismutases [20], photorespiration, and β-oxidation of fatty acids [18]. Hydrogen peroxide can generate more dangerous reactive oxygen species (ROS), such as the hydroxyl radical, through the Fenton reaction, and hypochlorous acid (HOCl) through the action of myeloperoxidase (MPO) in neutrophils [21]. These ROS can also react with bioactive molecules; for example, hydrogen peroxide oxidizes the thiol group of cysteine under stress conditions, glutathione by glutathione peroxidase [17], α-tocopherol [22], and β-carotene by the lactoperoxidase/H_2_O_2_ system [23], while hypochlorous acid deteriorates carotenoids [24] and γ-tocopherol [25]. Hypochlorous acid is a ROS produced biologically in appreciable amounts through the oxidation of the chloride ion, present in plasma (~ 100 mM), by H_2_O_2_ catalyzed with myeloperoxidase mainly in the neutrophils to protect against invaded bacteria and xenobiotics. HOCl is a potent oxidizing agent that can rapidly and non-enzymatically destroy biological molecules and trigger tissue damage [21].

α-Tocopheryl quinone (α-TQQ) is one of the primary oxidation products of α-tocopherol in vivo, formed from the degradation of α-tocopherol hydroperoxide, which, in turn, results from the reaction of α-TQ with singlet oxygen [26]. Although α-TQQ has no direct antioxidant activity, it can be reduced to α-TQ [27] or tocopheryl hydroquinones [28], and thus contributes to the overall cellular antioxidant potential. The oxidation process can take place in vivo by reactive oxygen species (ROS) e.g., singlet oxygen, peroxy radical, superoxide anion, or superoxide radical [26, 29] or in vitro under elevated temperature [30, 31]. α-TQQ was detected in various rat tissues and plasma (1–5 pmol/g or mL), with the concentration generally increasing under stress conditions [32].

Despite the crucial effects of hydrogen peroxide and hypochlorous acid as reactive oxygen species, information on identifying the oxidation products of tocopherols generated by these ROS and the biological activity of the resulting oxidation products is quite limited. Consequently, this work aims first to provide information on the oxidation of α-TQ by the two ROS, identify the oxidation products, and conduct a kinetic study that leads to the postulation of an oxidation mechanism. Second, the work examines the anti-breast and prostate cancer activities of α-TQ and the oxidation products.

## Materials and methods

### Chemicals

α-Tocopherol (α-TQ, (C_29_H_50_O_2_, MW 430.72)) was obtained from Sigma Co. Sodium hypochlorite solution (NaOCl, 15.18 mM), with available chlorine (4.0–6.0%, MW 74.44), was purchased from Advent Chembio Pvt.Ltd. Hydrogen peroxide (30%, 8.820 M) used was sourced from Research-Lab Fine Chem. MTT assay materials, breast (MCF-7) and prostate (PC-3) cancer cell lines, MTT dye and doxorubicin (Dox) were provided, and the assay was performed at the Science Way for Scientific Researches and Consultations Co.

### LC-ESI-MS^2^ analysis

Electrospray positive and negative ion acquisition modes (ESI-MS) were carried out on an XEVO-TQD triple quadruple instrument, Waters Corporation, Milford, MA 01757, U.S.A, mass spectrometer. The column was ACQUITY UPLC-BEH C18, 1.7 μm, 2.1 × 50 mm. The flow rate was 0.2 mL\min. The solvent system consisted of solvent A (aqueous 0.1% formic acid) and B (acetonitrile containing 0.1% formic acid). The gradient program was solvent A 90% (initial), 90% (2 min), 70% (5 min), 30% (15 min), 10 (22 min), 10 (25 min), 0% (26 min), 0% (29 min) and 90% (32 min). Mass source condition was voltage 3.0 kV and temperature 440 °C. Scan range 100–1000 m/z. Cone voltage 30 V and collision energy 20–30 eV.

### Kinetics of α-tocopherol oxidation

Hydrogen peroxide concentrations were determined spectrophotometrically at 230 nm using a molar absorptivity of 81 M^− 1^ cm^− 1^ [33]. HOCl was measured at 235 nm and pH 6 using a molar absorptivity of 100 M^− 1^ cm^− 1^ [34].

The kinetic parameters, including the rate constant and order of each reactant, were determined using the differential law approach [35]. The reaction rate was determined from the tangent slope of the [α-TQ] vs. time curve at the initial concentration decline. In the first series of experiments, the final concentration of hydrogen peroxide was fixed at 4.913 mM, and the α-TQ concentration was varied (73.533–330.900 µM). In the second series, the final concentration of α-TQ was constant at 147.07 µM, while the H_2_O_2_ concentration was in the range of 9.827–74.147 mM. Likewise, kinetics of oxidation by hypochlorous acid was performed using, in the first series, a final fixed HOCl concentration (533.33 µM) and a range of α-TQ concentrations (83.927-180.494 µM) in 3.0 mL; in the second series, the final α-TQ concentration was 208.530 and the HOCl concentration was in the range of 320.00-1600.00 µM. All experiments were performed in triplicate, and the reactions were monitored spectrophotometrically at the maximum absorbance of α-TQ (292 nm); concentrations were determined from the standard curve. The oxidation rate is expressed by the following rate law:

$$ - {\text{d}}[{\text{a}} - {\text{TQ}}]/{\text{dt}}\,=\,{\text{k }}[{\text{a}} - {\text{TQ}}\left] {^{{\text{x}}}} \right[{{\text{H}}_{\text{2}}}{{\text{O}}_{\text{2}}}{\text{or HOC}}{{\text{l}}^{\text{y}}}$$

where x and y are the kinetic order of α-TQ and oxidant, respectively, and k is the oxidation rate constant. Initial rates were obtained from the tangent slope of the initial decrease in α-TQ concentration with time. The reactant orders (x and y) and the rate constant (k) were derived from the linear regression of Ln (rate) vs. Ln (α-TQ concentration) according to the following equations:

$${\text{Ln }}( - {\text{d}}[{\text{a}} - {\text{TQ}}]/{\text{dt}})\,=\,{\text{Ln}}{{\text{k}}^\backslash }+{\text{ x Ln }}[{\text{a}} - {\text{TQ}}]$$

$$ \begin{gathered} {\text{Ln }}( - {\text{d}}[{\text{a}} - {\text{TQ}}]/{\text{dt}}) = \hfill \\ {\text{Lnk}}^{{\backslash \backslash }} + {\text{ y Ln }}\left[ {{\text{H}}_{{\text{2}}} {\text{O}}_{{\text{2}}} {\mkern 1mu} {\text{or HOCl}}} \right] \hfill \\ \end{gathered} $$

where

$$ {\text{Ln k}}^{\backslash } = {\text{ Ln k}}{\mkern 1mu} + {\mkern 1mu} {\text{y Ln }}\left[ {{\text{oxidant}}} \right], $$ and 

$$ {\text{Ln k}}^{{\backslash \backslash }} = {\text{ ln k}}{\mkern 1mu} + {\mkern 1mu} {\text{x Ln }}[{\text{a}} - {\text{TQ}}] $$

### Anticancer activity

The anticancer activity of α-TQ and its oxidation products was assessed against breast (MCF-7) and prostate (PC-3) cancer cells using the MTT assay [36]. Briefly, in a 96-well tissue culture plate, 10^5^ cells/ml (100 µL/well) in DMEM medium were incubated at 37 °C for 24 h to develop a complete monolayer sheet. Different concentrations of the oxidized product (0.1 mL) were added, leaving three wells as controls, and incubated at 37 °C for 24 h. Cells were checked for any physical signs of toxicity, e.g., partial or complete loss of the monolayer, rounding, shrinking, or cell granulation. Cells were washed twice with PBS solution to get rid of any excess of treating compounds that may react with MTT. The MTT solution (20 µL, 5 mg/mL in PBS) was added with shaking (5 min) to mix the dye in the medium. The plate was incubated for four hours at 37 °C to allow dye reduction mainly by the mitochondrial dehydrogenases. The medium was dumped, and the formazan crystal was dissolved in 200 µL DMSO with shaking for 5 min. The absorbance was measured at 560 nm with subtracting the background at 620 nm. The absorbance is directly proportional to the cell quantity. Data were analyzed by two-way ANOVA (SAS, v.9) to test the significant activity dependence on the test compound concentration and the difference between the means of α-TQ and its oxidation product effects.

## Results and discussion

### Identification of the α-tocopherol oxidation products

The physiological level of α-TQ depends on the organisms and tissues; it ranges from 20 to 60 µM [37]. Hydrogen peroxide is present in normal cells in concentrations of 1–5 µM [38] but can reach up to 50–600 µM in case of inflammation [38, 39]. The concentration of HOCl under physiological conditions can be up to 200 µM [40], but can reach 340 µM under various inflammatory diseases [41]. HOCl under normal conditions is crucial for the immunological system, but abnormal concentrations are related to oxidative stress diseases such as arthritis, kidney failure, atherosclerosis, Parkinson’s, and Alzheimer’s [40, 41]. Therefore, understanding the reaction mechanisms of these ROS, at high concentrations, with biological molecules, and identifying the structure of the oxidation products are of great value to human health.

Accordingly, the effects of H_2_O_2_ (4.91 mM) and HOCl (533.3 µM), at high concentrations on α-TQ (147.07 and 208.53 µM, respectively) for 96 and 24 h, respectively, were examined. Concentrations were chosen to give quantitative products in a reasonable time and yields that can be identified and examined for their biological activity. The oxidation products were subjected to the LC-ESI-MS^2^ analysis.

**Fig. 1:**
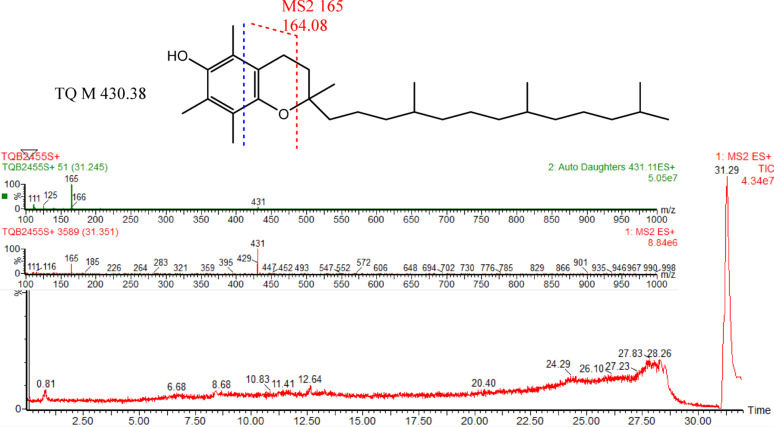
LC-MS-ESI chromatogram and spectra (MS^1^ and MS^2^ of fragment m/z 431) of α-TQ

**Fig. 2:**
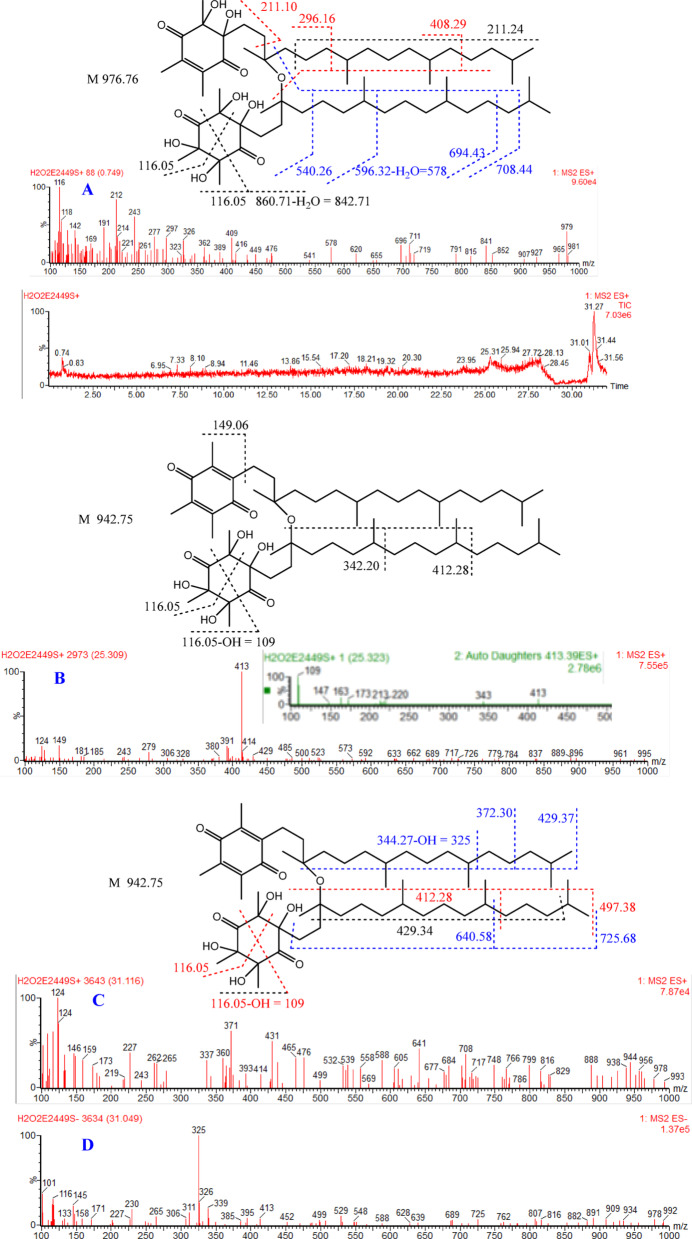
LC-MS-ESI chromatogram and spectra of α-TQ oxidation products by H_2_O_2_. (**A**) ES + is the spectrum of fraction at Rt 0.75 min, (**B**) ES + is the spectrum of fraction at Rt 25.3 min, and (**C**) and (**D**) are the ES + and ES- spectra, respectively of fraction at Rt 31.1 min

**Fig. 3:**
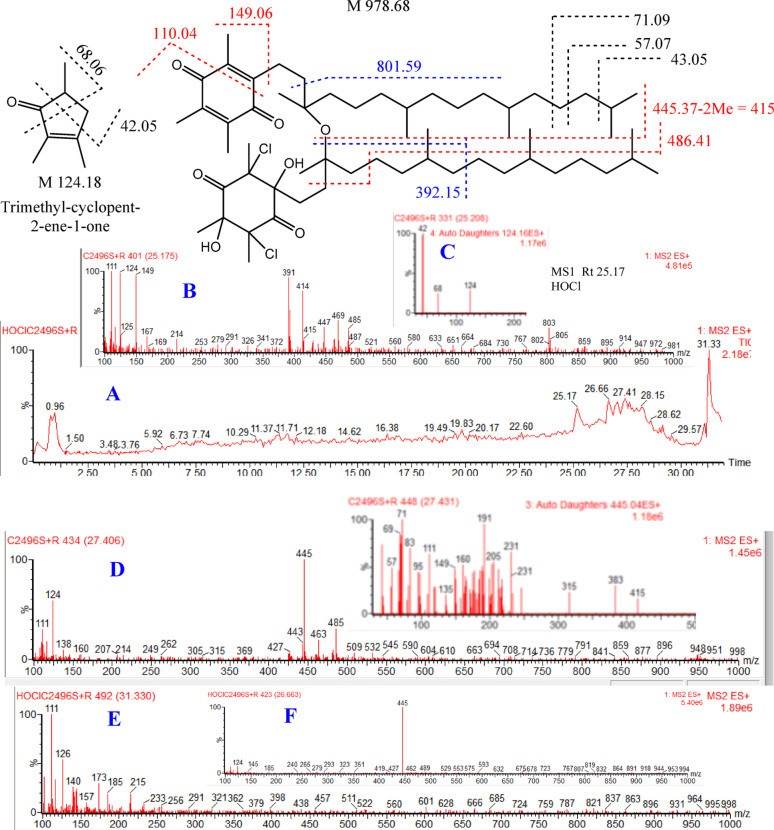
LC-MS-ESI chromatogram and spectra (ES+) of α-TQ oxidation products by HOCl. (**A**) is the chromatogram the oxidation products, (**B**) is the MS^1^ spectrum of fraction at Rt 25.18 min, (**C**) is the MS^2^ spectrum of the fragment at m/z 124 in spectrum (**B**), (**D**) is the MS^1^ spectrum of fragment at Rt 27.4 min and MS^2^ of the base peak at m/z 445, (**E**) MS^1^ of fraction at Rt 31.3 min, and (**F**) MS^1^ of fraction at Rt 26.7 min

The chromatogram of α-TQ (Fig. 1) indicates its purity, appearing at Rt 31.29 min. α-TQ eluted late in the chromatogram since it is a nonpolar compound and we used high polar solvent system (solvent A, aqueous 0.1% formic acid and B, acetonitrile containing also 0.1% formic acid) to allow a good separation of the expected more polar products which appeared in Figs. 2 and 3. α-TQ spectrum (ES+) showed that the molecular mass (M 430.38) is the base peak (M + 1 431) in addition to the M-1 peak (m/z 429). The fragment at m/z 165, which also appeared in the MS^2^ of the m/z 431 peak, is for the retro-Diels-Alder product (M 164.08) and a peak at m/e 111 for the indicated fragment (110.07). MS^2^ also showed a m/z peak at 125 for 2,3,5-trimethylcyclopent-2-en-1-one, which will be discussed later. Spectra of all oxidation products by H_2_O_2_ and HOCl showed high mass peaks (> 950), indicating dimerization.

The final products of α-TQ by H_2_O_2_ or HOCl resulted from oxidation to α-TQQ, followed by dimerization through ether linkage and the addition of one or more molecules of H_2_O_2_ or HOCl to the quinone’s four double bonds. The chromatograms of oxidation products (ES+) of H_2_O_2_ (Fig. 2) and HOCl (Fig. 3) showed mixtures of positional and stereoisomeric dimer additions, identified for the first time, while α-TQ and α-TQQ were not detected.

Few reports identified the dimer, mainly of γ-TQQ [42]. Ho et al. [43] observed the oxidation of γ-tocopherol model (2,2,7,8-tetramethyl-6-chromanol), which lacks the phytol group, by HOCl with electrophilic substitution of the chlorine atom on the aromatic ring and formation of a dichloro-pyrano-benzenedione. Nguyen and Southwell-Keely [25] also observed the electrophilic substitution of a chlorine atom on γ-tocopherol and detected the ether dimer of the chlorinated γ-TQQ. On the other hand, in the present research, α-TQ cannot undergo electrophilic substitution of the chlorine atom because of the full substitution of the aromatic ring, whereas, as discussed, the major products were the α-TQQ ether dimer and its addition adducts of the quinone double bonds, as illustrated in the proposed mechanism. It should be noted that all mentioned product identifications are putative MS/MS-based assignments and still need purification and NMR confirmation.

#### Identification of the H_2_O_2_ oxidation products

Figure 2 shows the spectra of the H_2_O_2_ oxidation reaction. The product at Rt 0.749 min gave the spectrum in Fig. 2A (ES+) with a molecular ion m/z 979 (M 976.79) resulting from the addition of H_2_O_2_ to three of the dimer double bonds. Peaks 212, 297, and 409 (depicted in red color) indicate the ring with mono H_2_O_2_ addition. The peak at 212 can also result from the alkyl side chain (C_16_H_33_) fragment. Peaks at 541, 578, 696, and 711 refer to the ring with two H_2_O_2_ additions (indicated in blue). The base peak at 116 also confirms the ring with two additions, which can result from two possible fragmentations of the ring.

The fraction at 25.309 min (ES+) is for an oxidation dimer with two H_2_O_2_ additions on one ring, as indicated by the spectral peaks (Fig. 2B) at m/z 124 and 149 for the unreacted ring; the m/z 124 peak results from the formation of the cyclopent-2-en-1-one ring, as will be confirmed later. The molecular ion (M 942.75) with water gives a peak at 961. The base peak at m/z 413 is a reliable indicator of the ring with two H_2_O_2_ additions, as its MS^2^ daughter spectrum exhibits a base peak at m/z 109 and a peak at m/z 343, as illustrated in the figure.

The fraction at 31.049 min is a diastereoisomer of the previous one (at 25.309 min). The unreacted ring is expressed by the spectral peaks (Fig. 2C, ES+) at m/z 124, 371, 431, 641 and 725 in addition to the base peak at m/z 325 in the ES- (Fig. 2D) spectrum while the ring with di-addition is manifested by the peaks at m/z 414 (ES+) and 413 (ES-) in addition to the peaks at m/z 499, 116 and 101 in both ES + and ES- spectra as illustrated in the figure.

The main difference between the spectrum of the dimer with three H_2_O_2_ additions at Rt 0.749 and the spectra of the two diastereoisomers with only two H_2_O_2_ additions on one ring is the absence of any fragment for an unreacted ring, e.g., m/z 124, 149, 325, 431, 641, and 725. In addition, the tri-addition adduct emerged earlier in the chromatogram due to its higher polarity.

#### Identification of the HOCl oxidation products

In the HOCl reaction chromatogram (ES+, Fig. 3A), peaks appeared after Rt 25.0 min have the major fragments of α-TQQ moiety (M 445), i.e., m/e 111 and 124, as well as peaks at 445 and/or 980, suggesting that α-TQQ is also the major and key product that can dimerize through ether linkage. The addition of HOCl to the double bonds is also indicated by the presence of M + 2 peaks of some fragments for the chlorine isotope (^37^Cl).

The product appeared at Rt 25.17 min, for example, gave major peaks at m/z 111 (100%), 124 (97.79%), 149 (94.60%), 414 (75.88%) and 485 (13.16%) for one of the quinone ring moieties of α-TQQ dimer as illustrated in Fig. 3B. The peak at m/z 124 is assigned for the trimethyl-cyclopent-2-ene-1-one, which was confirmed by its MS^2^ spectrum that contains m/z 42 and 68 fragments depicted in Fig. 3C. The 485 peak endorses the dimer form and the presence of one ring with no addition. Addition of two HOCl molecules to the second quinone ring is confirmed by peaks 391 (92.08%) and 803 (29.66%) in addition to their significant M + 2 peaks 393 (19.07%) and 805 (14.83%) respectively; besides, the molecular ion peak (M + 2H) at m/z 981 (1.5%) and its M + 2 peak at m/z 983 (0.91%).

Spectrum of peak at Rt 27.406 min (Fig. 3D) shows peaks of α-TQQ in dimer form i.e. 111 (29.78%), 124 (58.94%), 445 (100%) and 485 (30.92%) in addition to peaks at m/z 948 (M-2Me) and 998 (M + H_2_O) suggesting positional or diastereoisomer of previous compound (M 978.68). The structure α-TQQ fragment (m/z 445) was confirmed by the MS^2^ spectrum, which showed both peaks of the alkyl chain (m/z 43, 57, 71, and 83) and quinone ring (m/z 111, 149, and 191) in addition to a peak at m/z 415 for M-2Me as depicted in Fig. 3.

Fraction at 31.33 min could be confused with that of the original α-TQ peak (31.29 min) but it gave completely different spectrum (Fig. 3E) where the base peak of α-TQ (m/z 431) and the m/z 165 peak were not found; it gave a fragment at m/z 998 which might suggest to be isomer of previous compounds. The fraction at m/e 26.66 min yielded a base peak at 445 for the α-TQQ fragment, accompanied by the absence of a significant peak at higher masses, which renders further assignment uncertain (Fig. 3F).

### Effect of the pH on the rate of α-TQ oxidation by HOCl

**Fig. 4:**
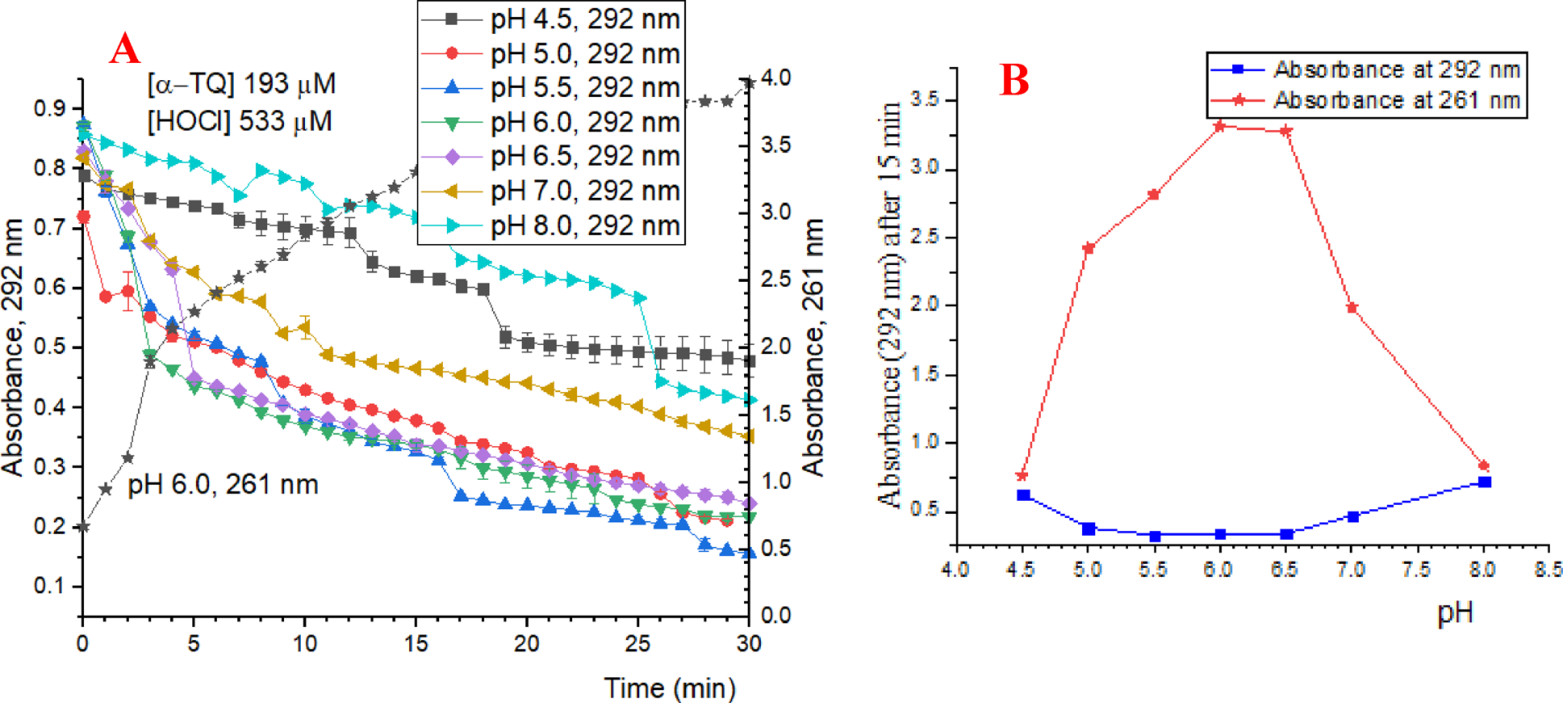
Effect of the pH on the rate of α-TQ oxidation by HOCl (**A**), and on the degradation of α-TQ at 292 nm and appearance of the oxidation products at 261 nm (**B**)

As mentioned above, oxidation by HOCl, as a strong ROS, is one of the main causes of the tocopherol loss in food and biological systems. The HOCl pKa is 7.5, and thus its composition (HOCl/OCl^−^) and oxidizing effect depend on the medium pH, which was found to vary in literature, 5-7.4 [21, 25, 44]. Therefore, the oxidizing effect of HOCl on α-TQ was examined under various pH (4.5-8.0) by following the absorbance of α-TQ at 292 nm and the reaction product, which was found to have maximum absorbance at 261 nm. Results presented in Fig. 4A and B showed that the maximum effect was found in the pH range 5.5–6.5. Accordingly, pH 6.2, which is also close to the physiological pH and corresponds to a HOCl: OCl^−^ ratio of 20:1, was chosen, as reported previously [34], to perform the α-TQ oxidation stability against HOCl.

### Kinetics of α-tocopherol oxidation

**Fig. 5:**
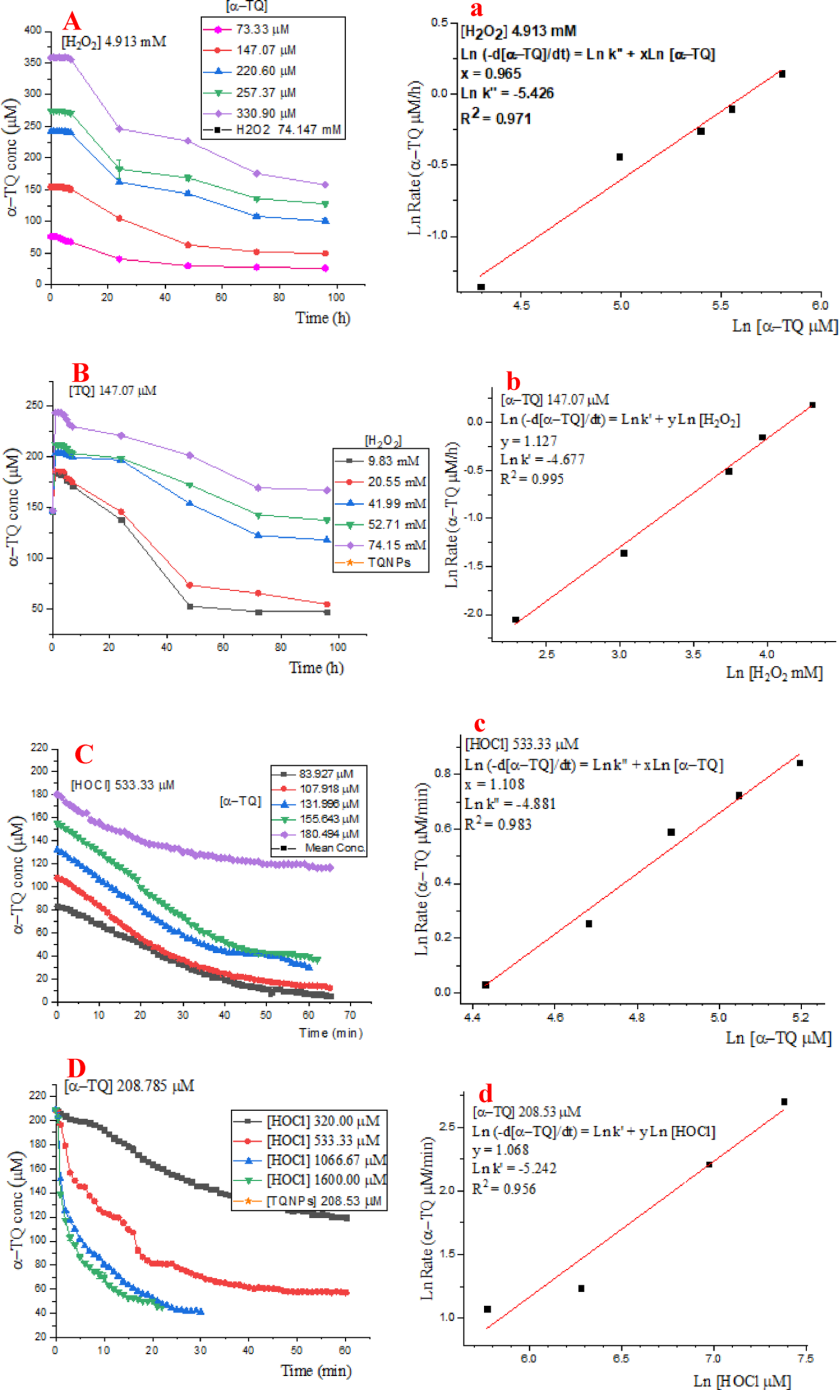
Kinetics of the α-TQ oxidation by H_2_O_2_ and HOCl (**A**–**D**) and the obtained linear regressions (a-d)

Reaction kinetics were inspected to shed some light on the α-TQ oxidation mechanism. The kinetic parameters were determined by the differential rate law approach [35], which allows determining the rate constant and the order of each reactant. A slight increase in absorbance and a few minutes of induction time were observed before the decline in α-TQ concentration; the initial rate is the tangent slope at the initial decline in the [α-TQ] vs. time curve. Two sets of experiments were designed, each with one reactant concentration kept constant and the concentration of the second reactant changed. Oxidation of α-TQ was found to be dependent on the concentrations of both α-TQ and the oxidant, either H_2_O_2_ or HOCl, as presented in Fig. 5A–D, respectively; subsequently, the differentiated rate law would be:

$$ - {\text{d}}[{\text{a}} - {\text{TQ}}]/{\text{dt}}\,=\,{\text{k }}{[{\text{a}} - {\text{TQ}}\left] {^{{\text{x}}}} \right[{{\text{H}}_{\text{2}}}{{\text{O}}_{\text{2}}}{\text{or HOCl}}]^{\text{y}}}$$

which could be converted, for the H_2_O_2_ oxidation reaction to the following linear equations:

$${\text{Ln }}( - {\text{d}}[{\text{a}} - {\text{TQ}}]/{\text{dt}})\,=\,{\text{Ln }}{{\text{k}}^\backslash }+{\text{ Ln }}[{\text{a}} - {\text{TQ}}]$$

$${\text{Ln }}( - {\text{d}}[{\text{a}} - {\text{TQ}}]/{\text{dt}})\,=\,{\text{Ln }}{{\text{k}}^{\backslash \backslash }}+{\text{ Ln }}\left[ {{{\text{H}}_{\text{2}}}{{\text{O}}_{\text{2}}}} \right]$$

where x and y are the order of α-TQ and H_2_O_2_, respectively, k is the rate constant.

$$ {\text{Lnk}}^{\backslash } = {\text{ Ln k}}{\mkern 1mu} + {\mkern 1mu} {\text{y Ln }}\left[ {{\text{H}}_{{\text{2}}} {\text{O}}_{{\text{2}}} } \right] $$ and 

$$ {\text{Ln k}}^{{\backslash \backslash }} = {\text{ Ln k}}{\mkern 1mu} + {\mkern 1mu} {\text{x Ln }}[{\text{a}} - {\text{TQ}}] $$

The linear correlations are presented in Fig. 5a–d for α-TQ oxidation by H_2_O_2_ and HOCl, respectively.

For both hydrogen peroxide and hypochlorous acid oxidations, the reaction is first-order in each of the α-TQ and the oxidant, suggesting similar reaction mechanisms as discussed below. However, the rate constant (Table 1) of α-TQ oxidation by HOCl (1.177 × 10^− 5^ µmol^− 1^ L^1^ min^− 1^) was much higher than that by H_2_O_2_ (6.301 × 10^− 7^ µmol^− 1^ L^1^ min^− 1^), i.e., 18.7-fold, revealing the severe adverse effect of the hypochlorous acid compared to that of hydrogen peroxide.

**Table 1:** Kinetic parameters of α-TQ oxidations by H_2_O_2_ and HOCl

Constant [H_2_O_2_] (4.913 mM µM)		Constant [α-TQ] (147.07 µM)		Rate Constant (k)	Order
[α-TQ] (µM)	Initial -d[α-TQ]/dt (µM/min)	[H_2_O_**2**_] (mM)	Initial -d[α-TQ]/dt (µM/min)		
Oxidation by H_2_O_2_					
73.533	15.480	9.827	7.740	k = 6.301 × 10^− 7^ µmol^− 1^ L^1^ min^− 1^	1st order in α-TQ, 1st order in H_2_O_2_
147.067	38.700	20.547	15.480		
220.6	46.440	41.987	36.120		
257.367	54.180	52.707	51.600		
330.900	69.660	74.147	72.240		

Constant [HOCl] (533.33 µM)		Constant [α-TQ] (208.785 µM)		Rate Constant (k)	Order
[α-TQ] (µM)	Initial -d[α-TQ]/dt (µM/min)	[HOCl] (µM)	Initial -d[α-TQ]/dt (µM/min)		
Oxidation by HOCl					
83.927	1.032	320.000	2.924	k = 1.177 × 10^− 5^ µmol^− 1^ L^1^ min^− 1^	1st order in α-TQ 1st order in HOCl
107.918	1.29	533.330	3.440		
131.996	1.806	1066.67	9.115		
155.643	2.064	1600.000	14.962		
180.494	2.322				

### Oxidative cleavage mechanisms

**Fig. 6:**
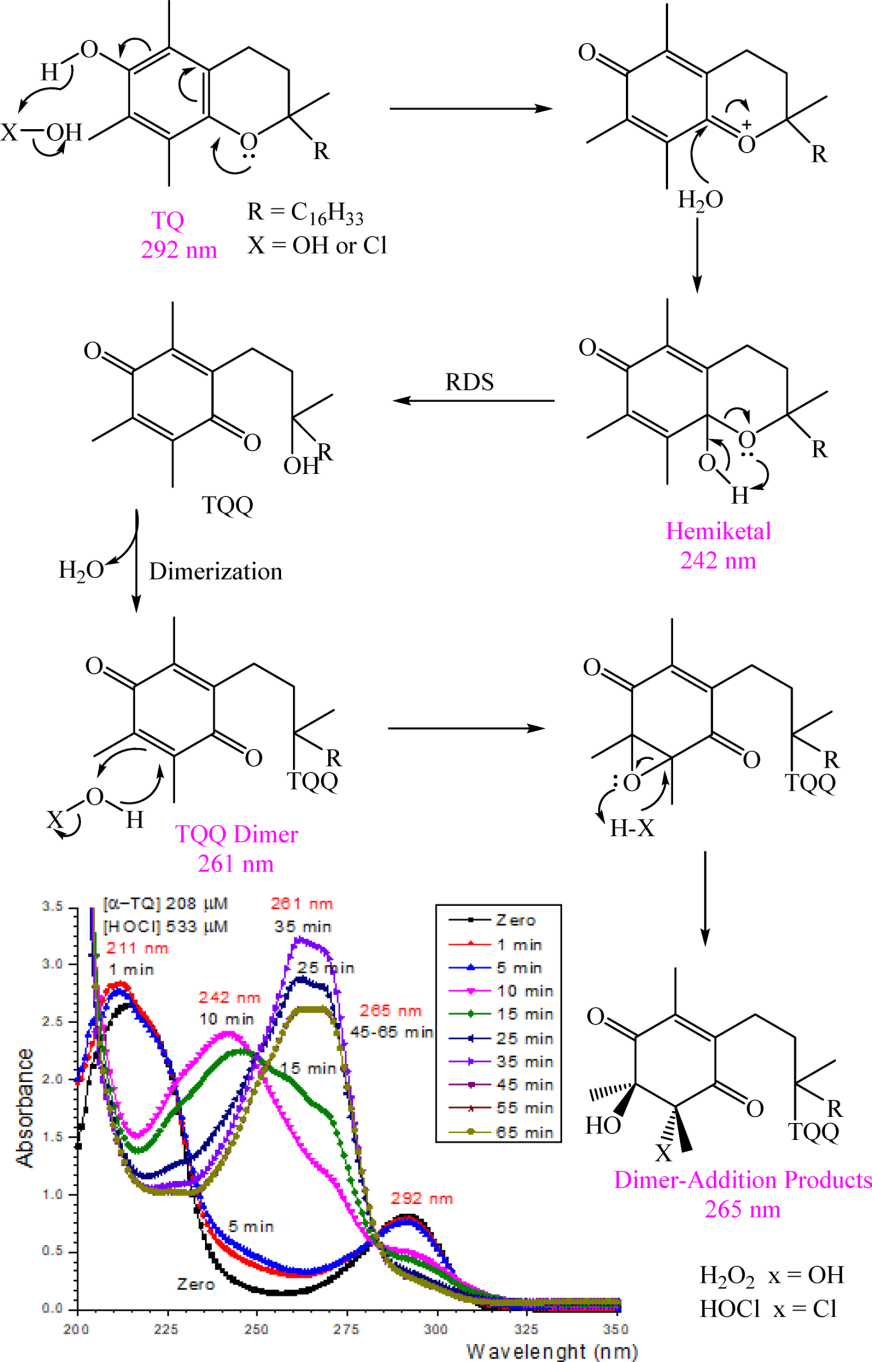
Mechanism of α-TQ oxidation by H_2_O_2_ or HOCl and UV-Vis spectra of α-TQ oxidation by HOCl at various intervals

Based on the identification of the reaction products and the kinetic behavior, the mechanisms of α-TQ oxidation by H_2_O_2_ and HOCl could be postulated as presented in Fig. 6. In addition to the kinetic similarity of the two reactions, the products of both oxidations showed that α-tocopheryl quinone (α-TQQ) is the key intermediate. The oxidation process takes place in the first step where a hydride ion is transferred to the oxidant, motivated by the resonance of the *p*-oxygen atom lone pair forming the oxonium quinone-like structure, which undergoes nucleophilic attack by a water molecule; the formed hemiketal (242 nm) is then rearranged with pyran ring opening to give the α-TQQ. The latter or its addition adducts of H_2_O_2_ or HOCl were not found in the reaction products, indicating that it undergoes fast dimerization through an ether linkage to give the TQQ-dimer (261 nm). The dimer undergoes addition of H_2_O_2_ or HOCl to the quinone double bonds, yielding a mixture of the identified products. The epoxidation of α, β-unsaturated carbonyl double bonds by hydrogen peroxide or hypochlorite is well-documented [45]. The rate-determining step (RDS) involves the oxidation step preceding the dimerization process, as the reaction exhibits first-order kinetics in both reactants. It can also be noted that the dimer has four double bonds, and the addition to each one creates two new chiral centers in addition to the formation of positional isomers, in the case of HOCl addition, which explains the observed large number of oxidation dimer products and the presence of many positional and diastereoisomers.

The oxidation reaction of α-TQ was also followed spectrophotometrically at various intervals; the α-TQ and HOCl concentrations were 208.785 and 533.33 µM, respectively. The obtained spectral series confirms the postulated mechanism in Fig. 6. The spectra of the reaction mixture, at the beginning (0–5 min), show the α-TQ spectrum with the characteristic peak at 292 nm while later (10–15 min), the 292 nm peak vanishes and a peak at 242 nm appears which is consistent with hemiketal structure. At 25 min, the latter peak disappears, and then a broad peak (261–270 nm) corresponding to α-TQQ emerges; it was reported that α-TQQ has a maximum absorbance at 262 nm [46]. The absorbance increases till 35 min; during this period (25–35 min), dimerization could take place since both α-TQQ and its dimer share the same chromogen and thus have similar maximum absorbance. Finally, the peak absorbance decreases (35–45 min) because of the occurrence of the addition reaction, giving a broad peak, with a slight bathochromic shift, centered at 265 nm, for the dimer-addition products.

It can be noted that α-TQQ is the major known oxidation product of α-TQ formed in vivo [47] and detected in oils [48]. Mechanistic studies have shown that the formation of the hemiketal and α-TQQ is a slow process [49, 50]. The 5-formyl-γ-tocopherol and epoxy α-tocopherol quinone (α-TQQ) were also identified [50]. α-TQQ was found to regulate the intestinal immune system and improve intestinal inflammation through the activation of the aryl hydrocarbon receptor and reduction of inflammation-inducing cytokine production [51]. α-TQQ also ameliorates liver fibrosis by enriching *Christensenella minuta* and regulating bile acid metabolism [52]. Other oxidation products were also detected, e.g., 13’-carboxychromanol and carboxyethyl hydroxychroman; the former was found to display anti-inflammatory activities while the latter was found to be the major αTQ metabolite in human urine [53].

### Anticancer activity of α-TQ and the oxidation products

Breast and prostate cancers are among the most common types of cancer affecting women [54] and men [55], respectively. In vivo experiments found α-TQA to be associated with the burden and metastases of breast cancer [56, 57]. α-TQ exhibited low activity, < 20% cell viability at 25 µM, against the positive estrogen-receptor MCF-7 breast cancer cell line but high activity, >90%, against the negative MDA-MB-231 at 25 µM [11] and 4T1 (40 µg/mL) breast cancer cell lines [57].

Meta-analysis indicated that α-TQ, not γ-TQ, blood level was inversely related to prostate cancer development [58]. α-TQ showed a reduction of less than 20% and 45% at concentrations of 10 and 50 µM, respectively, in the proliferation of androgen-dependent PC-3 prostate cancer cells [59]. α-TQ combined with naringenin showed a synergistic effect on ornithine decarboxylase expression in the PC-3 prostate cancer cell line [55].

Data regarding the anticancer activity of α-TQQ, the initial metabolite product of tocopherol oxidation, are limited and yield also varying results based on the tested cancer cells, while the activity of the α-TQQ dimer has not been investigated. For instance, α-TQQ exhibited good activity against the androgen-responsive prostate cancer cell lines (LAPC4 and LNCaP), i.e.,>50% cell growth inhibition at 40 µM, while α-TQ showed only < 25% at the same concentration; meanwhile, both tocopherols had no activity towards the androgen-independent DU145 prostate cancer cells. α-TQQ was found to inhibit the androgen-induced activation and the release of prostate-specific antigen from the carcinogenic cells [60]. In addition, tocopheryl quinones exhibited potential anticancer activities; γ-TQQ induces ~ 60% and 25% apoptosis in myeloid (promyelocytic) leukemia HL-60 and colon adenocarcinoma WiDr cells, respectively, after four hours at concentrations of 50 and 200 µM, respectively [61]. α-TQQ also showed toxicity against the CEM, drug-sensitive lymphoblastic leukemia cells (24%), and the CEM/VLB100, multidrug-resistant lymphoblastic leukemia cells (14%), whereas α-TQ did not affect either cell type [62]. Additionally, α-TQQ displayed activity with an IC50 of 8.5 µM against colon cancer (HCT116 cells), whereas the IC50 of α-TQ was >100 µM [63].

**Fig. 7:**
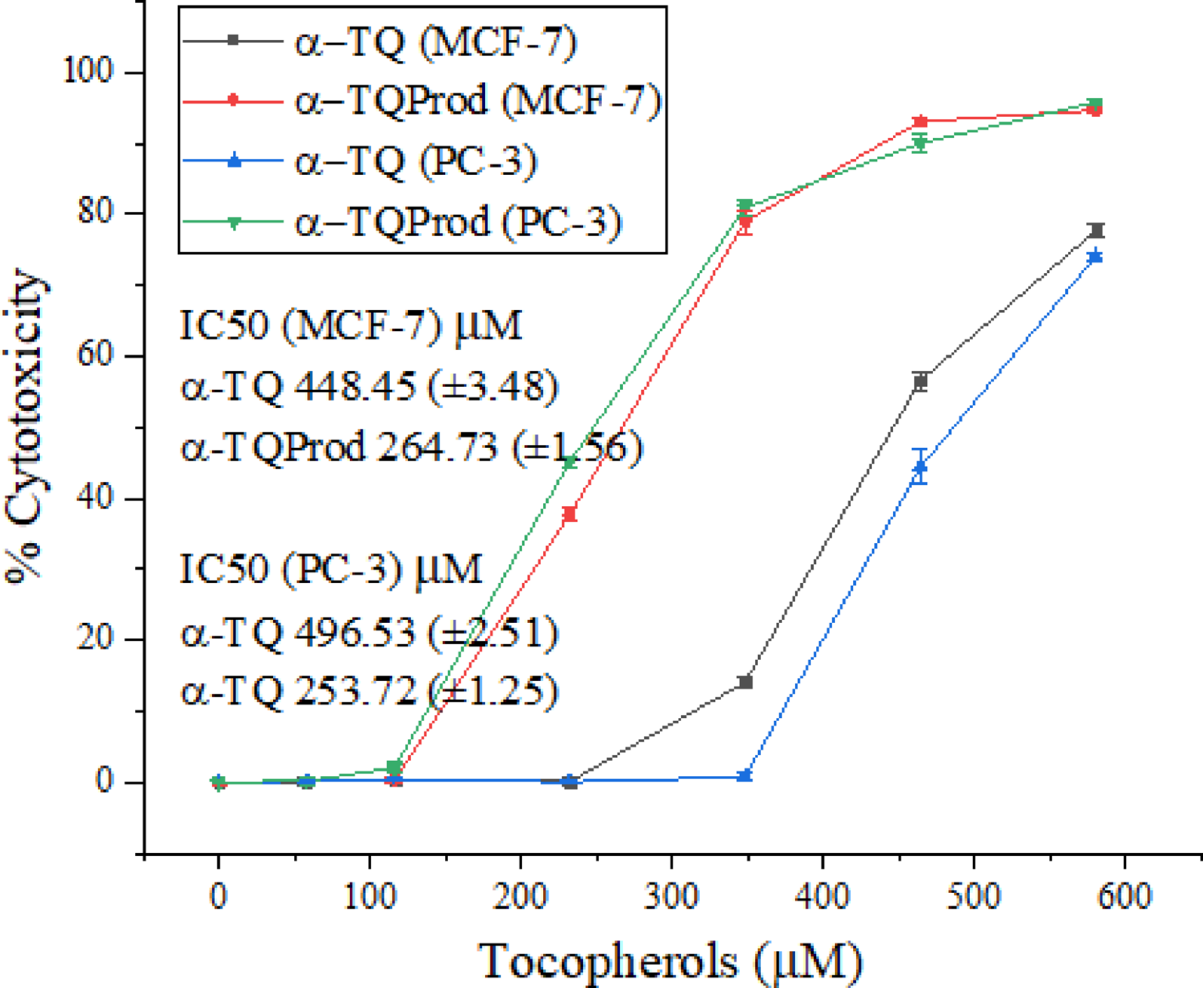
Cytotoxicity of α-TQ and α-TQQ oxidation dimer products against breast (MCF-7) and prostate (PC-3) cancer cells

Accordingly, the α-TQ and α-TQQ dimer products, resulting from H_2_O_2_ oxidation, were examined against estrogen-dependent breast cancer (MCF-7) and androgen-dependent prostate cancer (PC-3) cells. The results (Fig. 7 and Table S1) revealed that α-TQ was inactive up to 232.18 µM and 348.26 µM against MCF-7 and PC-3, respectively; thereafter, the toxicity steadily and significantly increased to 77.69% and 74.06%, respectively, at 580.44 µM. These findings might explain the previously observed low activity at low concentrations against both cell lines in the reports mentioned above [11, 59].

On the other hand, the α-TQQ dimers of the α-TQ oxidation products (α-TQProd) also exhibited no activity at low concentrations, but only up to 116.09 and 58.04 µM against MCF-7 and PC-3, respectively; then started to increase significantly, reaching 94.65 and 95.82% toxicity, respectively, against both cells at a concentration of 580.44 µM. Statistical analysis (Table S1) also showed that the increase in cytotoxicity against both cell lines by α-TQQ dimers was significantly higher than that of α-TQ at *p* ≤ 0.05. The higher activity of α-TQQ dimers was also reflected by the lower IC50 values against both cell lines (264.73 and 253.72 µM, respectively) than those of α-TQ (448.45 and 496.53 µM, respectively).

The anticancer activity of tocopheryl quinones can be attributed to either the reduction of quinones through the redox cycle, resulting in the formation of reactive oxygen species (ROS), or the nucleophilic attack of proteins or DNA on quinones as a Michael acceptor [62].

## Conclusion

α-TQ is severely affected by H_2_O_2_ and HOCl oxidation. The oxidation products were identified by LC-MS/MS as a dimer of the initial oxidation product, α-TQQ, through an ether linkage. The quinone double bonds undergo H_2_O_2_ or HOCl addition, resulting in the formation of several positional and diastereoisomers. Kinetically, the reactions are found to follow a first-order profile in each of α-TQ and the oxidant. Oxidation by HOCl depends on the medium pH, with a maximum rate at pH 5.5–6.5, which is close to the physiological pH. The similarity in product profiles and kinetic behaviors of the two oxidant reactions suggests similar oxidation mechanisms. The reaction was followed spectrophotometrically, where the intermediates, hemiketal and α-TQQ, absorbed at 242 and 261 nm, respectively, while the dimer addition final products absorbed at 265 nm.

α-TQ showed cytotoxicity against breast and prostate cancer at concentrations > 232.18 and 348.26 µM, reaching 77.69% and 74.06% at 580.44 µM with IC50 of 448.45 and 496.53 µM, respectively. Conversely, α-TQQ exhibited anticancer activity at concentrations > 116.09 and 58.04 µM, reaching cytotoxicity of 94.65% and 95.82% at 580.44 µM with IC50s of 264.73 and 253.72 µM, representing 1.7- and 2.0-fold higher toxicity than that of α-TQ towards breast and prostate cancer, respectively. These results indicate that natural α-TQ and its oxidation products, when administered at a suitable dose, can provide protection against various types of cancer. However, the obtained information is limited to in vitro MTT data and requires further validation.

## Supplementary Information

Below is the link to the electronic supplementary material.

Supplementary Material 1.

## Data Availability

All data related to the present work are included in the MS.

## Abbreviations

α-TQ: α-Tocopherol
α-TQQ: α-Tocopheryl quinone
α-TQProd: α-Tocopherol oxidation products
ROS: Reactive oxygen species
