# Supplementary material for: Oxidative degradation of alpha-tocopherol by reactive oxygen species, identifying products, and product anticancer activity
Source: BMC Chem. 2025 Nov 15;19(1):306. doi: 10.1186/s13065-025-01665-1 (PMC12619437; doi:10.1186/s13065-025-01665-1)
Supplement: Supplementary file 1 — Supplementary Material 1. [file 13065_2025_1665_MOESM1_ESM.docx]

**Oxidative Degradation of Alpha-Tocopherol by Reactive Oxygen Species, Identifying Products, and Product Anticancer Activity**

**Supplementary Materials**

**Table S1.** Cytotoxicity of α-TQ and α-TQQ oxidation dimer products against breast (MCF-7) and prostate (PC-3) cancer cells

| **MCF-7** | **α-TQ**  **% Toxicity (± SD)** | **IC50**  **µM (± SD)** | **α-TQ Product**  **% Toxicity (± SD)** | **IC50**  **µM (± SD)** |
| --- | --- | --- | --- | --- |
| **α-TQ (µM)** |  |  |  |  |
| 0.0 | 0.0 (± 0.274) | 448.446  (± 3.483) | 0.0 (± 0.274) | 264.726  (± 1.556) |
| 58.044 | 0.091 (± 0.619)^f^ |  | 0.229 (± 0.210)^f^ |  |
| 116.087 | 0.229(± 0.210)^f^ |  | 0.274 (± 0.412)^f^ |  |
| 232.175 | 0.091 (± 0.482)^f^ |  | 37.631 (± 0.913)^d^ |  |
| 348.262 | 14.083 (± 0.780)^e^ |  | 78.967 (± 1.693)^b^ |  |
| 464.35 | 56.562 (± 1.318)^c^ |  | 93.050 (± 0.554)^a^ |  |
| 580.437 | 77.686 (± 1.100)^b^ |  | 94.650 (± 0.363)^a^ |  |
| **PC-3** | **α-TQ**  **% Toxicity (± SD)** | **IC50**  **µM (± SD)** | **α-TQ Product**  **% Toxicity (± SD)** | **IC50**  **µM (± SD)** |
| **α-TQ (µM)** |  |  |  |  |
| 0.0 | 0.0 (± 0.242) | 496.529  (± 2.507) | 0.0 (± 0.242) | 253.721  (± 1.254) |
| 58.044 | 0.139 (± 0.279)^f^ |  | 0.232 (± 0.564)^f^ |  |
| 116.087 | 0.325 (± 0.213)^f^ |  | 1.999 (± 0.702)^f^ |  |
| 232.175 | 0.046 (± 0.213)^f^ |  | 45.095 (± 0.688)^e^ |  |
| 348.262 | 0.744 (± 0.564)^f^ |  | 80.893 (± 1.142)^c^ |  |
| 464.35 | 44.351 (± 2.467)^e^ |  | 90.005 (± 1.296)^b^ |  |
| 580.437 | 74.059 (± 0.639)^d^ |  | 95.816 (± 0.418)^a^ |  |

α-TQ Product concentration was calculated as α-TQ. Values sharing the same letter in each cell line are insignificantly different at p≤0.05
